# Parents’ perceived supervisor support and work-to-family enrichment affect job and family satisfaction in dual-income parents and their adolescents

**DOI:** 10.3389/fpsyg.2026.1694200

**Published:** 2026-05-19

**Authors:** Berta Schnettler, Andrés Concha-Salgado, Ligia Orellana-Calderón, Mahia Saracostti, Katherine Beroíza, Héctor Poblete, Germán Lobos, Cristian Adasme-Berríos, María Lapo, Leonor Riquelme-Segura, José A. Sepúlveda

**Affiliations:** 1 Facultad de Ciencias Agropecuarias y Medioambiente, Universidad de La Frontera, Temuco, Chile; 2 Centro de Excelencia en Psicología Económica y del Consumo, Universidad de La Frontera, Temuco, Chile; 3 Scientific and Technological Bioresource Nucleus (BIOREN-UFRO), Universidad de La Frontera, Temuco, Chile; 4 Universidad Católica de Santiago de Guayaquil, Guayaquil, Ecuador; 5 Departamento de Psicología, Universidad de La Frontera, Temuco, Chile; 6 Departamento de Trabajo Social, Universidad de Chile, Santiago, Chile; 7 Facultad de Economía y Negocios, Universidad de Talca, Talca, Chile; 8 Departamento de Economía y Administración, Universidad Católica del Maule, Talca, Chile; 9 Departamento de Trabajo Social, Universidad de La Frontera, Temuco, Chile

**Keywords:** adolescents, cross-domain effect, dual-income parents, supervisor support, within-domain effect, work-to-family enrichment

## Abstract

**Introduction:**

Drawing on the Conservation of Resources theory and the work-family enrichment framework, this study posits that resources acquired in the workplace, such as supervisor support, generate benefits that enhance outcomes both within the work domain and across the family domain. While work-to-family enrichment (WtoFE) is a recognized driver of job and family satisfaction, research exploring how these resources cross over to affect the well-being of both partners and their adolescent children remains limited. The research explored both the direct and indirect relationships, as well as the relationships between individuals, related to perceived supervisor support, WtoFE, and job and family satisfaction among dual-income parents and their adolescent children.

**Methods:**

Using a cross-sectional, single-timepoint design, 430 parents who both earn an income and have at least one adolescent child were recruited for the research through non-probabilistic sampling. The mediation Actor-Partner Interdependence Model and structural equation modeling were employed for data analysis.

**Results and Discussion:**

The results indicate that perceived supervisor support from both parents enhances job satisfaction but does not equally affect family satisfaction. Notably, fathers’ support boosts mothers’ job satisfaction and adolescents’ family satisfaction. This support also contributes to parents’ WtoFE, positively influencing both family and job satisfaction. Mothers’ support specifically enhances fathers’ WtoFE, while fathers’ support benefits mothers’ and adolescents’ family satisfaction. Both parents’ WtoFE plays a mediating role between their perceived supervisor support and family and job satisfaction at individual and inter-individual levels. These findings underscore the importance of developing strategies that enhance supervisor support to improve job satisfaction for parents and family satisfaction for all family members.

## 1 Introduction

Many employees juggle paid jobs with family obligations, a dynamic traditionally associated with conflict (Yucel and Minnotte, 2017). However, this intersection also involves the acquisition of resources (Landolfi et al., 2020) through work-family enrichment, where one role improves the quality of life in another (Greenhaus and Powell, 2006). Central to this synergy is the Conservation of Resources (COR) theory (Hobfoll, 1989), which posits that individuals strive to obtain and protect resources to manage life’s demands. While work-family enrichment is a reciprocal process, this study concentrates on work-to-family enrichment (WtoFE). We prioritize this direction because workplace resources are often more susceptible to external organizational influence, making the transfer from work to home a critical intervention point for family well-being (Greenhaus and Powell, 2006).

Following the COR perspective, workplace support acts as a critical contextual resource (ten Brummelhuis and Bakker, 2012). Among various sources of support, we focus specifically on perceived supervisor support—the belief that a supervisor values an employee’s contributions and cares about their well-being (Shanock and Eisenberger, 2006). Theoretically, supervisors function as primary “resource gatekeepers”; they directly control the allocation of social and instrumental resources that spark the enrichment process (Ollier-Malaterre et al., 2020). Consequently, this study examines job and family satisfaction as key consequences of perceived supervisor support and WtoFE. Job satisfaction is the extent to which employees find pleasure in their work (Agho et al., 1992). On the other hand, family satisfaction involves a conscious, individual assessment of one’s family life (Zabriskie and Ward, 2013).

Within the enrichment literature, Zhang et al. (2018) propose that the work-family enrichment model is the most systematic framework for understanding both within-domain and cross-domain effects. Within-domain effects pertain to the relationships between enrichment and outcomes within the same domain from which the resources arise, such as the impact of WtoFE on job satisfaction (Amstad et al., 2011). In contrast, cross-domain effects occur when WtoFE influences the family domain, specifically affecting family satisfaction (Frone et al., 1992). By exploring both perspectives, this study provides new insights into how WtoFE influences satisfaction across domains, expanding our understanding beyond the individual employee’s experience, which remains somewhat limited.

In this regard, enrichment literature has predominantly concentrated on the individual perspective, with limited research on inter-individual experiences or crossover effects (Agrawal and Mahajan, 2021). Crossover involves the transfer of resources among individuals who share a similar environment, such as family members (Bakker and Demerouti, 2013; Chen et al., 2015). This gap is important because the flow of resources influences not only the employee’s well-being (actor effect) but also their family members through a crossover effect (partner or interindividual effect). Specifically, this study addresses a unique theoretical gap by emphasizing the contribution of including adolescents in the analysis of family dynamics, as they are highly sensitive to their parents’ work-life quality (Matias and Recharte, 2021). Furthermore, most research has been conducted in Western or Asian regions (Agrawal and Mahajan, 2021), leaving a gap in Latin American contexts such as Chile, where couples and families may experience the work-family interface in distinct ways (Ho et al., 2013).

To address these gaps, the general objective of this study is to examine both the direct and indirect individual and interindividual associations among perceived supervisor support, work-to-family enrichment, and job and family satisfaction among Chilean dual-income parents and their adolescents. This research offers three primary contributions. First, it evaluates the robustness of within-domain versus cross-domain effects using a mediation Actor-Partner Interdependence Model (APIM; Kenny et al., 2006). Second, it extends the enrichment literature by demonstrating how workplace resources transcend the couple to affect the well-being of adolescent children. Finally, it provides empirical evidence from a Latin American context, expanding the understanding of the work-family interface beyond Western and Asian perspectives by exploring how these dynamics operate in a culture with distinct gender role expectations and family structures.

The transfer of resources within the dyad may also be shaped by Gender Role Theory (Eagly and Wood, 2012). Societal expectations regarding motherhood and fatherhood can influence how resources are invested and received between partners. By applying an APIM framework grouped by gender, this study acknowledges that the ‘resource caravan’ may flow differently depending on whether the source is the mother or the father, providing a more nuanced understanding of how gendered dynamics influence both the partner and the adolescent children.

We employed a comparable approach to evaluate the direct and indirect impacts of a job demand (specifically, workload) on both job and family satisfaction through work-to-family conflict. This study employed a sample with similar characteristics (Schnettler et al., 2025) and was carried out under the guidelines of the APIM. This model examines how the effects of individuals (actors) and their partners could be affected by the traits of the dyad and the dynamics of their relationships (Garcia et al., 2015).

### 1.1 Hypotheses development

Research indicates that support from a supervisor can have a positive influence on work outcomes (Carlson et al., 2019). According to COR theory (Hobfoll, 1989), supervisor support acts as a contextual resource that minimizes the drain of personal energy, allowing employees to maintain a positive reservoir of resources. Consistent with this, in a sample of bank employees in Indonesia, Burhanudin et al. (2020) reported that supervisor support is positively associated with job satisfaction. Similar findings were reported by Kalliath et al. (2020) among social workers in Australia, Otu et al. (2021) among Nigerian police officers, Öksüz et al. (2023) among employees in the Turkish hospitality industry, and Le et al. (2025) among employees in travel agencies in Vietnam.

**Figure 1:**
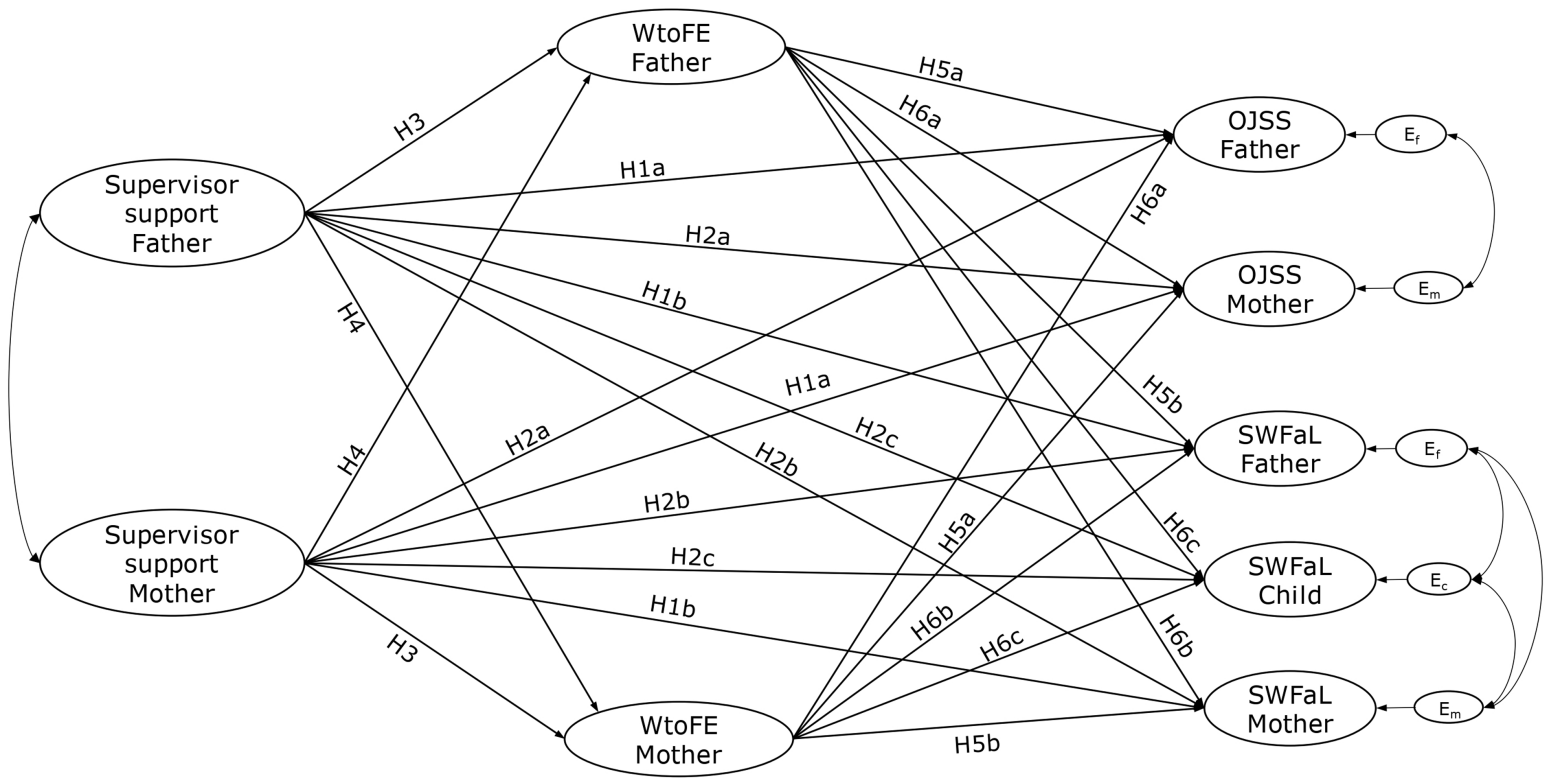
Conceptual framework illustrating the anticipated relationships between Perceived Supervisor Support, Work-to-Family Enrichment (WtoFE), Job Satisfaction (OJSS), and Family Satisfaction (SWFaL) among dual-income parents and their adolescent children. Em, Ec, and Ef: residual errors on OJSS and SWFaL for mothers, adolescent children, and fathers, respectively. The conceptual path diagram excluded the indirect effects of WtoFE (H7 and H8) to maintain clarity in the figure.

Furthermore, the COR framework suggests that resources are not static; they can be transferred from the work domain to improve life in the family domain. A positive work environment has been linked to more time dedicated to home activities and interactions with children, enhanced quality of engagement with family members, and increased family satisfaction (Frone et al., 1995). Nevertheless, the specific direct connection between supervisor support and family satisfaction has been studied very little. Drummond et al. (2017) found a positive connection between supervisor support and family satisfaction among female employees, as well as among employees from China and Hong Kong. Therefore, we posited the first hypothesis (Figure 1):

*H1*: The perceived support from each parent’s supervisor is positively associated with both (a) their job satisfaction and (b) their family satisfaction.

Regarding crossover effects between members of a couple, the evidence is scarce. Theoretically, within the COR framework, this relationship emerges through a crossover process in which the resource gain of one individual reduces their stress and increases their emotional availability, thereby enriching their partner’s resource pool (Bakker and Demerouti, 2013). For instance, Booth-LeDoux et al. (2020) discovered that when workers feel supported in the workplace, their levels of burnout decrease. This positive change is also noticed by their partners at home. As a result, partners feel that the employee is better able to offer emotional support, allowing them to dedicate more resources to their work. Ultimately, this may lead to increased job satisfaction for the partners (Soomro et al., 2018).

In line with this “resource investment” logic, workplace resources can be transferred from one partner to another, thereby enhancing the recipient’s resources and positively impacting the family domain. This transfer can strengthen marital and family bonds, as well as improve parental experiences (Brady et al., 2021). Steiner and Krings (2016) emphasized that supervisors’ social support may be a crucial factor in an employee’s work-family enrichment, positively impacting their spouse’s well-being and overall family functioning. Additionally, in a study involving dual-income parents with adolescents, Schnettler et al. (2022b) reported that fathers’ perceived workplace support was positively associated with the diet quality of mothers and their adolescent children in Chile. This suggests that the “resource caravan” (Hobfoll, 2011) initiated by a supervisor’s support transcends the individual to benefit the entire family unit. Thus, the second hypothesis posited (Figure 1).

*H2*: The perceived support from one parent’s supervisor is positively associated with (a) the job satisfaction of the other parent, (b) the family satisfaction of the other parent, and (c) the family satisfaction of the adolescents.

WtoFE starts with the resources available in the work environment. The primary theoretical lens for understanding this connection is the COR theory (Hobfoll, 2001), which posits that individuals possess a finite pool of resources and are motivated to engage in behaviors—such as utilizing workplace support—to acquire additional resources. According to Hobfoll’s perspective, when a supervisor provides support, they are not just facilitating a task; they are providing a contextual resource that an employee can then “re-invest” in their family role. In a meta-analytic study comprising 171 independent studies, Lapierre et al. (2018) reported a positive association between supervisor support and work-family enrichment (both directions of enrichment). Carlson et al. (2019) also found that supervisor support is positively associated with the employee’s WtoFE. Similar results were reported by Kalliath et al. (2020) and Burhanudin et al. (2020). Therefore, we posited the third hypothesis (Figure 1).

*H3*: The perceived support a parent receives from their supervisor is positively linked to their work-to-family enrichment.

Extending this COR logic to the dyadic level, research indicates that workplace support can also have positive effects on partners. Specifically, Hobfoll (2011) describes that resources do not exist in isolation but in “caravans”; thus, the support one partner receives at work creates a surplus of energy and psychological capital that can cross over to enhance the other partner’s ability to navigate their own work-family enrichment. For instance, in China, Ho et al. (2013) found that when husbands feel socially supported at work, it has a positive impact on the work-family enrichment their wives experience. More recently, a study by Brady et al. (2021) also suggests that the support one partner perceives at work may contribute to improved work-to-family enrichment for the other partner. Therefore, we argued that similar results may be possible for supervisor support in particular and posed the fourth hypothesis (Figure 1).

*H4*: The support one parent receives from their supervisor is positively linked to the work-to-family enrichment of the other parent.

The “resource caravan” concept introduced by Hobfoll (2011) suggests that when individuals perceive support, it can initiate a positive cycle of resource gains that boosts WtoFE. This process of gaining additional resources via work-family enrichment is expected to improve both job satisfaction and family satisfaction subsequently.

Consistent with the within-domain effect, a meta-analytic research with 67 studies by Zhang et al. (2018) discovered a favorable correlation between WtoFE and job satisfaction. Theoretically, while COR theory explains that the work domain is the primary source of the resources that enable enrichment (Hobfoll, 2001), the within-domain perspective suggests that this leads to an affective reaction toward the work role; specifically, individuals feel more satisfied with their jobs because they perceive them as the origin of the benefits experienced in their family life (Amstad et al., 2011). This logic is supported by Xu et al. (2018) in another meta-analytic study that encompassed the results of more than 30 countries with varying levels of development and individualism. More recently, some researchers have reported similar findings, such as Burhanudin et al. (2020) in Indonesia.

Parallelly, consistent with the cross-domain effect, it has been demonstrated that resources acquired in a person’s workplace can influence their experiences in the family sphere (Carlson et al., 2019). Theoretically, the COR framework explains this as a resource transfer where the “psychological capital” gained at work provides the necessary energy to enhance family interactions. In this regard, Zhang et al. (2018) reported a positive relationship between WtoFE and family satisfaction. Other studies that have found a positive relationship between WtoFE and family satisfaction include those of Landolfi et al. (2020) and Lo Presti et al. (2020) with Italian employees, Orellana et al. (2022) with Chilean dual-income couples, as well as the meta-analysis carried out by Xu et al. (2018). Thus, the fifth hypothesis proposed a positive association between WtoFE and job and family satisfaction among parents (Figure 1).

*H5*: The work-to-family enrichment of each parent is positively linked to both their (a) job and (b) family satisfaction.

Expanding this logic to the family system, WtoFE can create resources and establish a robust “resource reservoir,” which is crucial for enhancing performance (Greenhaus and Powell, 2006), not only for the employee but also for their partner. Within the COR framework, this reservoir facilitates a crossover process in which the enrichment of one parent reduces the family’s overall systemic stress, thereby serving as a resource gain for the partner (Hobfoll, 2011). Following the within-domain logic, once the partner receives these resources, they experience a more positive affective state, which enhances their job satisfaction, as they perceive their environment as more supportive and resourceful. In this regard, Liu and Cheung (2015) reported that wives’ WtoFE was positively linked to their husbands’ job satisfaction in a Chinese sample of dual-income couples.

Research carried out in the European Union suggests crossover effects of WtoFE among family members. In a study involving Italian dual-income couples, Lo Presti et al. (2020) found that one partner’s WtoFE has a positive influence on their partner’s family satisfaction. Notably, this “resource caravan” also extends to children. Theoretically, COR theory suggests that when parents bring resources from work into the home, they increase the family’s collective energy and reduce conflict, creating a more nurturing environment for child development (Hobfoll, 2011). Consistently, Matias and Recharte (2021) discovered that the WtoFE of Portuguese mothers positively influences the well-being of their adolescent children. Research by Schnettler et al. (2022a) on Chilean dual-income couples with adolescents reported that both parents’ WtoFE indirectly influences their well-being in the food domain and that of their adolescent children by fostering a positive perception of the family meal atmosphere. More recently, a meta-analysis study by Bilodeau et al. (2023) found that parents’ work-family enrichment is related to children’s positive mental health. Thus, the sixth hypothesis posited (Figure 1).

*H6*: A parent’s work-to-family enrichment is positively associated with (a) the job satisfaction of the other parent, (b) the family satisfaction of the other parent, and (c) the family satisfaction of adolescents.

The fundamental concept of the cross-domain effect (often referred to as the domain specificity model) and the within-domain effect (also known as the source attribution perspective) suggests that WtoFE acts as a bridge between work and family (Shockley and Singla, 2011). From the perspective of COR theory, this mediation represents a “resource investment” chain: an initial contextual resource (supervisor support) provides the necessary conditions for employees to develop new personal resources (WtoFE), which are then reinvested to achieve job satisfaction via source attribution and family satisfaction via resource transfer (Hobfoll, 2001). Therefore, we suggest that WtoFE mediates between perceived supervisor support and job satisfaction, as well as between perceived supervisor support and family satisfaction. Lu and Chang (2014) found that WtoFE mediates between supervisor support and job satisfaction in a sample of Chinese employees. Similar results were reported by Burhanudin et al. (2020). Ollier-Malaterre et al. (2020) reported that WtoFE acts as a link between family-supportive organizational perceptions and job burnout in various countries. Furthermore, this mediating role extends beyond the individual level. Carlson et al. (2019) discovered that WtoFE serves a mediating role at both individual and interindividual levels, acting as a bridge between the support workers receive from their supervisors and both their marital satisfaction and that of their partners.

Similarly, this interindividual mediation is crucial for the family unit in the Latin American context. Schnettler et al. (2022b) found that WtoFE mediates between a job resource and a performance outcome at both individual and interindividual levels. The authors reported that for mothers, WtoFE mediates the relationship between their perceived workplace support for families and their diet quality. For fathers, their perception of workplace support for families mediates the relationship between that support and the diet quality of their adolescent children. These findings suggest that WtoFE is the primary mechanism through which the “resource caravan” initiated by the supervisor reaches all family members. Therefore, we posited the seventh and eighth hypotheses, which involve individual and interindividual relationships.

*H7*: The WtoFE mediates the association between parents’ perceived supervisor support and job satisfaction.

*H8*: The WtoFE mediates the association between parents’ perceived supervisor support and the three family members’ family satisfaction.

Finally, some research has explored the relationship between WtoFE and both family and job satisfaction simultaneously. From a COR perspective, resources are most potent within their domain of origin, as they are contextually tailored to the roles that generated them (Hobfoll, 2001). This is consistent with the within-domain perspective, which posits that employees develop a stronger affective reaction toward the work environment as the source of their enrichment (Amstad et al., 2011), leading to a more robust correlation with job satisfaction than with family satisfaction.

In this regard, Lu and Chang (2014) only found support for the within-domain approach when studying the associations between WtoFE and job and family satisfaction, specifically that WtoFE was significantly related only to job satisfaction. More recently, a meta-analysis conducted by Zhang et al. (2018) revealed a significant correlation between WtoFE and both job and family satisfaction. Notably, the association was significantly stronger for job satisfaction compared to family satisfaction, supporting the premise that resource reinvestment and affective attribution are most efficient within the source domain. Similarly, the meta-analysis conducted by Xu et al. (2018) revealed that the association between WtoFE and job satisfaction was more significant than the link between WtoFE and family satisfaction, across various countries with differing degrees of individualism and economic development. Therefore, although both meta-analyses (Xu et al., 2018; Zhang et al., 2018) provide support to both approaches, based on the theoretical logic of resource proximity and the empirical strength of the within-domain relationship over the cross-domain relationship, we formulated the last hypothesis:

*H9*: The connection between WtoFE and job satisfaction is considerably stronger than the connection between WtoFE and family satisfaction.

## 2 Methods

### 2.1 Sample

The criteria for selecting participants in this study included parents of different sexes who were dual-income earners (either in a marriage or living together) and had a minimum of one adolescent child between the ages of 10 and 15 years. Participants were recruited using a convenience non-probability sampling method, with allocations determined by the socioeconomic distribution of households in Temuco, Chile, classified into three socioeconomic categories: high, medium, and low. This strategy was implemented to ensure a varied representation of socioeconomic backgrounds. The final sample comprised 430 dual-income families. This sample size exceeds current recommendations for mediation models within the APIM, which suggest a minimum of 91 dyads to detect actor effects and 249 dyads to detect partner effects (Ledermann et al., 2022). Therefore, the present sample can be considered adequate to ensure sufficient statistical power for the analyses conducted and to reflect an effort to capture greater heterogeneity in the representation of Chilean families.

### 2.2 Measures

Parents were requested to fill out the following scales:

The Perceived Supervisor Support Scale (Karasek et al., 1985) consists of ten items, such as: “My supervisor is concerned about the welfare of those under him or her”. Participants evaluated the items using a 4-point Likert scale, where 1 represents “not at all” and 4 signifies “very much.” This study utilized the Spanish version of the scale, which has been tested with Chilean workers (Schnettler et al., 2023). The scores from the Perceived Supervisor Support Scale are derived by adding together the responses from the ten items, with higher totals reflecting a greater perception of support from supervisors.

The Overall Job Satisfaction Scale (OJSS), developed by Agho et al. in 1992, includes six items, such as: “I feel fairly well satisfied with my job”. Participants indicated their agreement with each statement using a five-point Likert scale, where 1 represents “strongly disagree” and 5 represents “strongly agree.” The study utilized a validated Spanish version of the OJSS, which has demonstrated strong internal consistency in research related to dual-income couples in Chile (Schnettler et al., 2024, 2025). Scores were derived by summing the responses from the six items, with higher totals reflecting a greater degree of job satisfaction.

The Nijmegen Work-Home Interaction Survey [SWING; Wagena and Geurts, 2000; as referenced in Kinnunen et al. (2006)] was utilized to assess work-to-family enrichment. This involved three items from the related subscale that focus on the positive impact of work on family life, particularly in terms of mood, skills, and behavior (for instance, “You fulfill your domestic obligations better because of the things you learned in your job?”). Participants evaluated each item using a 5-point scale, where 1 indicates “never” and 5 signifies “very often.” A validated Spanish version of this measure was utilized, which has shown robust internal consistency in research involving dual-income parents in Chile (Orellana et al., 2022; Schnettler et al., 2022a). The total score is calculated by adding the three items, with higher scores indicating greater levels of WtoFE.

The subsequent scale was given for completion by mothers, fathers, and adolescents:

The Satisfaction with Family Life Scale (SWFaL, Zabriskie and Ward, 2013) is a five-item measurement derived from the Satisfaction with Life Scale (Diener et al., 1985). In this scale, the word “life” is substituted with “family life” in each of the five statements. For example, one statement says, “I am satisfied with my family life”. Participants provide their ratings on a scale from 1 (strongly disagree) to 6 (strongly agree). This study utilized the validated Spanish version of the scale, which has demonstrated internal consistency in research conducted with Chilean adults and adolescents (Orellana et al., 2022; Schnettler et al., 2024, 2025). Scores are calculated by summing the five items, with higher totals representing greater satisfaction with family life.

Mothers, fathers, and adolescents were asked about their age, with adolescents also asked to indicate their gender. Mothers and fathers were questioned about their weekly working hours and the nature of their work arrangements. Mothers were asked explicitly about the size of their family and the quantity of children they had. The family’s socioeconomic status (SES) was determined based on the total household income, household size, and the education and occupation of the primary income earner in the household (Agencia de Investigación para el Mercado, 2024).

### 2.3 Procedure

Families were reached through their adolescents’ schools and social networks. Trained interviewers clarified the study’s objectives and the questionnaire format to the parents, assuring them that their responses would remain confidential and anonymous. Families that consented to participate provided the email address of a family member, usually the mother. This email was utilized to send the online questionnaire links to each family member. Interviewers subsequently made follow-up phone calls to address any inquiries and confirm that the questionnaires were completed. Data collection occurred from June to November 2024. The response rate was 90.2%.

At the start of the online questionnaire, mothers and fathers were provided with a consent form, while adolescents received an informed assent form. Each family member confirmed their willingness to participate by ticking a box. The finalized questionnaires were kept in separate databases on the QuestionPro platform (QuestionPro Inc.). After all three family members completed their questionnaires, they were compensated with a $10 bank transfer.

We introduced two proactive quality control strategies to assess the quality of completed questionnaires. First, we provided participants with compensation for the time they spent filling out the survey. This monetary incentive was designed to promote engagement and increase the chances of receiving thoughtful, high-quality responses. Second, at the beginning of each response section, we provided clear instructions on how to complete each scale and included a neutral example to illustrate the expected response style. This method ensured that participants fully understood the format and requirements of the survey items.

Forty families took part in a pilot test that utilized the same recruitment strategy and data collection methods without needing any modifications. This study is part of a larger project examining the connections among work, family, and food-related demands and resources in Chilean households (Schnettler et al., 2023). The Ethics Committee of Universidad de La Frontera has approved the study (protocol number 035-23).

### 2.4 Data analysis

IBM SPSS Statistics 23 (IBM Corp., Armonk, NY, USA) was used to perform descriptive analysis. The Perceived Supervisor Support Scale has not been previously used in dyadic analysis within the Spanish language context. To address this gap, a dyadic confirmatory factor analysis (CFA) was performed, following the methodology proposed by Claxton et al. (2015), to investigate its latent structure and psychometric properties. The Omega coefficient was used to measure the internal consistency of the scale (McDonald, 1970). Convergent validity was evaluated by examining the standardized factor loadings of the scale, which should ideally be above 0.5, alongside their statistical significance and the average variance extracted (AVE), with values greater than 0.5 being desirable (McDonald, 1970).

After evaluating the psychometric properties of the supervisor support measure, and prior to hypothesis testing, common method variance (CMV; Chang et al., 2010) was examined by conducting Harman’s single-factor test separately for mothers and fathers. All items from the measured constructs –the Perceived Supervisor Support Scale, the Overall Job Satisfaction Scale, Work-to-family enrichment, and the Satisfaction with Family Life Scale– were loaded into a factor analysis. This procedure identifies whether a single factor is prominent or if multiple factors are present, with one potentially explaining most of the covariance among the four measures. According to Chang et al. (2010), CMV is not a concern if no single factor explains the majority of the covariance. The analysis was conducted in SPSS using principal component analysis (PCA) without rotation.

To evaluate Hypotheses 1–8, this study employed the mediation Actor-Partner Interdependence Model (APIM) in conjunction with structural equation modeling (SEM), following the methodology proposed by Kenny et al. (2006). The research focused on the relationships among variables for individual family members (actor effects) as well as the interactions between family members (interindividual, crossover, or partner effects). In this context, both fathers and mothers were seen as both actors and partners, whereas adolescents were regarded solely as partners. The analysis examined the relationships between parents’ perceptions of supervisor support, WtoFE, and job satisfaction, as well as satisfaction of family members with their family.

The APIM facilitates understanding of the connections among family members. Following the methodology described by Kenny et al. (2006), the APIM examined the correlations (covariances) between each parent’s perception of supervisor support. The APIM also includes correlations among the residual errors of the dependent variables, particularly the parental variables (work-to-family enrichment and job satisfaction), alongside those of the three members of the family (family satisfaction). According to the authors, these correlations contribute to recognizing other causes of interdependence within each dyadic relationship.

The analysis also incorporated factors that have a direct effect on the dependent variables for mothers and fathers, specifically work-to-family enrichment and job satisfaction, as well as overall family satisfaction among all three family members. This adjustment was made to statistically account for external influences that might affect the connections between the main variables in the model. The included variables were the ages of the three family members, the workplace conditions, the family’s socioeconomic status, and the number of children.

Mplus 8.11 (Muthén & Muthén, Los Angeles, CA, USA) was utilized for conducting the CFA and SEM. The Unweighted Least Squares Mean and Variance adjusted (ULSMV) estimator was chosen, given that the data consist of Likert-type items with an ordinal nature. This robust estimation method relies on polychoric correlation matrices, allowing for a more appropriate modeling of the latent structure underlying ordered categorical variables. Furthermore, with a sample size of 430 triads, ULSMV achieves adequate performance in parameter stability and accuracy, reducing potential bias in parameter estimates and standard errors that may arise when normality assumptions are violated. The adequacy of the CFA and SEM models was determined by the Tucker-Lewis index (TLI) and the comparative fit index (CFI). A TLI or CFI value exceeding 0.95 indicates a strong fit, while a value over 0.90 reflects an acceptable fit. Additionally, the root mean square error of approximation (RMSEA) was used, where values under 0.06 signify a good fit and those under 0.08 suggest an acceptable fit (Hu and Bentler, 1999).

Using the approach recommended by Lau and Cheung (2012), SEM was employed to evaluate the mediating role of work-to-family enrichment (Hypotheses 7 and 8). This involved applying a bias-corrected (BC) bootstrap confidence interval, utilizing 1,000 samples. Confidence intervals that do not include zero suggest the presence of a mediating effect.

The path coefficients were evaluated using SEM to compare the differences between the association of work-to-family enrichment with job satisfaction (within-domain perspective) and work-to-family enrichment with family satisfaction (cross-domain perspective). This analysis was conducted at both individual and interindividual levels for each parent, examining the relationships between mothers and fathers, as well as those between parents and their adolescent children (Hypothesis 9).

## 3 Results

All data were complete, with no missing entries. Table 1 showcases the sociodemographic characteristics of the sample. All data was complete, with no missing entries. The study involved 430 families, comprising mothers, fathers, and adolescents, resulting in a total of 1,290 family members surveyed. The majority of households were of middle socioeconomic status, comprising four family members, including two children. Most parents worked at their job sites.

**Table 1:** Demographic characteristics of the sample.

Characteristic	Total sample (*n* = 430)
Age [Mean (*SD*)]	
Mother	37.6 (6.8)
Father	40.5 (8.5)
Adolescent	12.6 (1.7)
Adolescents’ gender (%)	
Male	50.5
Female	49.5
Number of family members [Mean (*SD*)]	4.2 (0.9)
Number of children [Mean (*SD*)]	2.1 (1.1)
Socioeconomic status (%)	
High	17.4
Middle	80.9
Low	1.6
Mothers’ place of work (%)	
Remote	3.3
In-person	87.0
Mixed	9.8
Fathers’ place of work (%)	
Remote	0.2
In-person	92.3
Mixed	7.5

Table 2 presents the mean scores and connections for parents’ perceived supervisor support (SS), work-to-family enrichment (WtoFE), job satisfaction (OJSS), and family satisfaction (SWFaL) of three family members. All observed correlations were statistically significant and consistent with the anticipated patterns. There were no significant differences between fathers and mothers regarding scores on perceived supervisor support (*t* = 1.730, *p* = 0.084), WtoFE (*t* = −0.923, *p* = 0.356), and OJSS (*t* = 0.858, *p* = 0.235). However, mothers reported significantly lower scores than fathers and adolescents in SWFaL (*F* = 13.965, *p* < 0.001). Fathers did not display a difference compared to adolescents.

**Table 2:** Summary statistics and correlation analysis for Perceived Supervisor Support (SS), Work-to-Family Enrichment (WtoFE), Job Satisfaction (OJSS), and Family Satisfaction (SWFaL) in different-sex dual-income parents and their adolescent children (*n* = 430).

Variable	Correlations									
	*M* (SD)	1	2	3	4	5	6	7	8	9
1. Mothers’ SS	22.6 (6.0)	1	0.404***	0.313***	0.237***	0.363***	0.144**	0.197***	0.172***	0.137**
2. Fathers’ SS	21.9 (6.2)		1	0.204***	0.236***	0.250***	0.390***	0.143**	0.136**	0.147**
3. Mothers’ WtoFE	10.7 (2.7)			1	0.301***	0.273***	0.107*	0.251***	0.146**	0.116*
4. Fathers’ WtoFE	10.8 (2.6)				1	0.146**	0.207***	0.203***	0.214***	0.148**
5. Mothers’ OJSS	21.2 (4.5)					1	0.299***	0.219***	0.214***	0.148**
6. Fathers’ OJSS	20.8 (4.8)						1	0.222***	0.286***	0.155**
7. Mothers’ SWFaL	23.2 (4.3)							1	0.648***	0.515***
8. Fathers’ SWFaL	24.3 (4.4)								1	0.485***
9. Adolescents’ SWFaL	24.8 (4.3)									1

*p < 0.05, **p < 0.01, *** *p* < 0.001.

### 3.1 Psychometric properties of the perceived supervisor support scale

The initial dyadic CFA of the Perceived Supervisor Support Scale did not adequately fit the data, as items 3 and 7 (both reversals) exhibited factor loadings below 0.1 (please refer to the items included in the Supplementary material). Consequently, a second CFA was carried out, excluding these items. The results from this analysis showed that the measurement model of the scale fits the data well for both parents (RMSEA = 0.035, CFI = 0.989, TLI = 0.986). The analysis also showed that the scale demonstrates strong reliability, with Omega coefficients of 0.96 and 0.97 for mothers and fathers, respectively. All factor loadings were statistically significant (*p* < 0.001), indicating strong convergent validity (mothers’ range = 0.822–0.909, fathers’ range = 0.835–0.923). The AVE was 0.77 and 0.78 for mothers and fathers, respectively.

### 3.2 Assessing common method variance

According to Harman’s single-factor test, the PCA results from the four measures utilized in this study indicated the presence of four distinct factors for both mothers and fathers, accounting for 73.5 and 72.8% of the total variance, respectively. The first unrotated factor, corresponding to the Perceived Supervisor Support Scale, explained only 34.8% of the variance for mothers and 34.5% for fathers. Overall, no single factor emerged as dominant, and the first factor did not capture the majority of the variance in either group, suggesting that CVM is not a concern in this study (Chang et al., 2010).

### 3.3 APIM findings: examining both individual and interindividual hypotheses

All standardized factor loadings for the scales used were statistically significant (*p* < 0.001) and exceeded 0.50, confirming convergent validity. The AVE values were strong: mothers’ perceived supervisor support = 0.83, fathers’ perceived supervisor support = 0.84, mothers’ WtoFE = 0.57, fathers’ WtoFE = 0.52, mothers’ OJSS = 0.66, fathers’ OJSS = 0.66, mothers’ SWFaL = 0.80, fathers’ SWFaL = 0.78, and adolescents’ SWFaL = 0.77. The omega coefficients demonstrated adequate to high level of reliability across scales: mothers’ perceived supervisor support = 0.97, fathers’ perceived supervisor support = 0.97, mothers’ WtoFE = 0.80, fathers’ WtoFE = 0.76, mothers’ OJSS = 0.91, fathers’ OJSS = 0.92, mothers’ SWFaL = 0.95, fathers’ SWFaL = 0.94, and adolescents’ SWFaL = 0.94.

**Figure 2:**
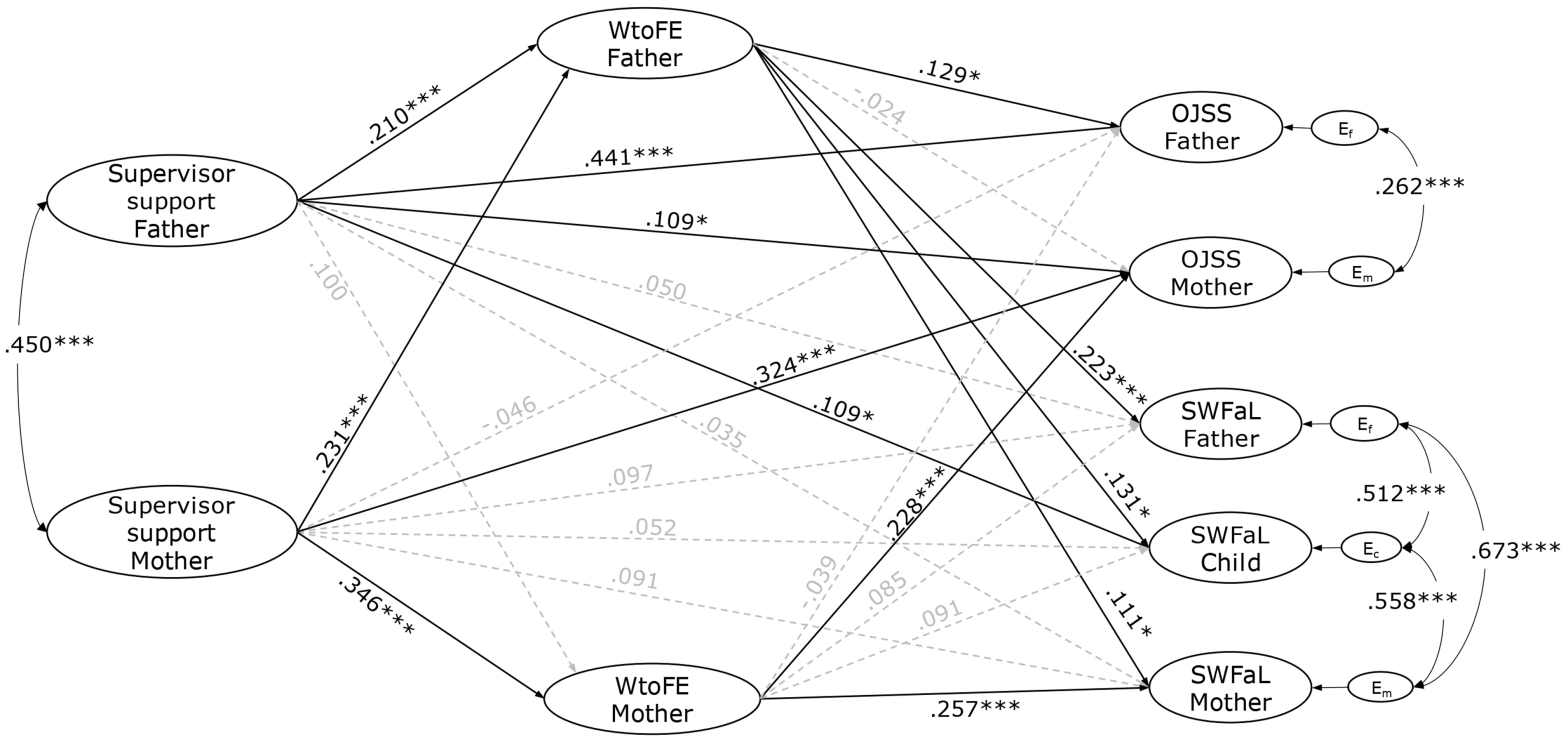
Actor-partner interdependence model of the effect of Perceived Supervisor Support, Work-to-Family Enrichment (WtoFE), Job satisfaction (OJSS), and Family Satisfaction (SWFaL) in dual-income parents and their adolescent children. E_m_, E_c_, and E_f_: residual errors on OJSS and SWFaL for mothers, adolescent children, and fathers, respectively. *p < 0.05. **p < 0.01. ***p < 0.001. It does not show the correlation between the residual errors of parents’ WtoFE and the effect of the family SES, the number of children, the ages of the three family members, as well as both parents’ weekly working hours on the parents’ WtoFE and OJSS, and the SWFaL of all three family members.

The results from the structural model estimation are shown in Figure 2. The APIM model examined the relationships between mothers’ and fathers’ perceived supervisor support, work-to-family enrichment, job satisfaction, and the family satisfaction of the three family members. The model demonstrated an excellent fit with the data (CFI = 0.991; TLI = 0.990; RMSEA = 0.011). A significant correlation was identified between parents’ perceived supervisor support (*r* = 0.450, *p* < 0.001). Additionally, significant correlations emerged between the residual errors of mothers’ and fathers’ WtoFE (*r* = 0.287, *p* < 0.001), OJJS (*r* = 0.262, *p* < 0.001), and SWFaL (*r* = 0.673, *p* < 0.001), as well as between mothers’ and adolescents’ SWFaL (*r* = 0.558, *p* < 0.001) and between fathers’ and adolescents’ SWFaL (*r* = 0.512, *p* < 0.001).

The ages of the three family members and the family’s SES had a significant impact on the model as control variables. The mothers’ age negatively affected their own (*β* = −0.159, *p* = 0.028), the fathers’ (*β* = −0.162, *p* = 0.030), and the adolescents’ (*β* = − 0.196, *p* = 0.008) SWFaL. The adolescents’ (*β* = −0.166, *p* = 0.001), and the fathers’ (*β* = −0.218, *p* = 0.004) ages negatively affected the adolescents SWFaL. Lastly, the family’s SES had a positive influence on the mothers’ SWFaL (*β* = 0.113, *p* = 0.046).

The standardized path coefficients, as depicted in Figure 2, demonstrate a significant association between both fathers’ (*β* = 0.441, *p* < 0.001) and mothers’ perceived supervisor support (*β* = 0.324, *p* < 0.001) and their OJSS. This finding supports hypothesis H1a for both mothers and fathers. In contrast, neither fathers’ (*β* = 0.050, *p* = 0.309) nor mothers’ (*β* = 0.091, *p* = 0.073) perceived supervisor support showed a significant relationship with their SWFaL. Therefore, these results do not support hypothesis H1b.

Fathers’ perceived supervisor support was positively associated with the mothers’ OJSS (*β* = 0.109, *p* = 0.025). In contrast, mothers’ perceived supervisor support was not significantly related to fathers’ OJSS (*β* = −0.046, *p* = 0.370). These results supported H2a only for fathers. Neither the fathers’ perceived supervisor support was significantly related to the mothers’ SWFaL (*β* = 0.035, *p* = 0.462) nor the mothers’ perceived supervisor support was significantly linked to the fathers’ SWFaL (*β* = 0.097, *p* = 0.076). These findings did not support H2b. The fathers’ perceived supervisor support was positively related to the adolescents’ SWFaL (*β* = 0.109, *p* = 0.033), whereas the mothers’ perceived supervisor support was not significantly associated with the adolescents’ SWFaL (*β* = 0.052, *p* = 0.310). These results partially supported H2c.

Fathers’ (*β* = 0.210, *p* < 0.001) and mothers’ (*β* = 0.346, *p* < 0.001) perceived supervisor support was positively related to their WtoFE; thus, H3 was supported for mothers and fathers. Whereas mothers’ perceived supervisor support was positively associated with the fathers’ WtoFE (*β* = 0.231, *p* < 0.001), fathers’ perceived supervisor support was not significantly associated with the mothers’ WtoFE (*β* = 0.100, *p* = 0.078). Therefore, these findings supported H4 only for fathers.

Fathers’ (*β* = 0.129, *p* = 0.022) and mothers’ (*β* = 0.228, *p* < 0.001) WtoFE were positively associated with their OJSS. Similarly, both fathers’ (*β* = 0.223, *p* < 0.001) and mothers’ (*β* = 0.257, *p* < 0.001) WtoFE showed a positive relationship with their SWFaL. These results support H5a and H5b for both mothers and fathers.

Neither the fathers’ WtoFE was statistically associated with the mothers’ OJSS (*β* = −0.024, *p* = 0.643), nor was the mothers’ WtoFE related to the fathers’ OJSS (β = −0.039, *p* = 0.475). These results did not support H6a for fathers and mothers. The fathers’ WtoFE was positively linked to the mothers’ SWFaL (*β* = 0.111, *p* = 0.047); however, the mothers’ WtoFE was not statistically linked to the fathers’ SWFaL (*β* = 0.085, *p* = 0.139). Thus, these findings supported H6b only for mothers. Whereas the fathers’ WtoFE was positively associated with the adolescents’ SWFaL (*β* = 0.131, *p* = 0.026), the mother’s WtoFE was not significantly related to the adolescents’ SWFaL (*β* = 0.091, *p* = 0.093). These results supported H6c only for fathers.

### 3.4 The role of work-to-family enrichment as a mediator

The research also investigated how parents’ WtoFE influences the link between perceived supervisor support and job satisfaction for parents (H7), as well as between parents’ perceived supervisor support and family satisfaction among the three family members (H8). The results indicated that fathers’ WtoFE mediates the relationship between their perceived supervisor support and their job satisfaction, as shown through a significant indirect effect (Table 3). A similar finding was observed concerning the association between mothers’ perceived supervisor support and fathers’ job satisfaction. The mediating effect of mothers’ WtoFE was found to be significant only in the relationship between their perceived supervisor support and job satisfaction. These results partially confirmed H7.

**Table 3:** Bias-corrected confidence intervals of specific mediation effects of parents’ work-to-family enrichment (WtoFE).

Specific indirect effects	Estimate	Lower 2.5%	Upper 2.5%	*p*-value
Fathers’ SS → Mothers’ WtoFE → Fathers’ OJSS	0.004	−0.007	0.015	0.495
Fathers’ SS → Fathers’ WtoFE → Fathers’ OJSS	0.027	0.001	0.053	0.041*
Mothers’ SS → Mothers’ WtoFE → Fathers’ OJSS	0.013	−0.024	0.051	0.482
Mothers’ SS → Fathers’ WtoFE → Fathers’ OJSS	0.030	0.002	0.058	0.037*
Fathers’ SS → Mothers’ WtoFE → Mothers’ OJSS	0.023	−0.005	0.050	0.105
Fathers’ SS → Fathers’ WtoFE → Mothers’ OJSS	−0.005	−0.027	0.017	0.648
Mothers’ SS → Mothers’ WtoFE → Mothers’ OJSS	0.079	0.036	0.122	< 0.001***
Mothers’ SS → Fathers’ WtoFE → Mothers’ OJSS	−0.006	−0.026	0.019	0.648
Fathers’ SS → Mothers’ WtoFE → Fathers’ SWFaL	−0.009	−0.007	0.024	0.271
Fathers’ SS → Fathers’ WtoFE → Fathers’ SWFaL	0.047	0.013	0.081	0.007**
Mothers’ SS → Mothers’ WtoFE → Fathers’ SWFaL	0.030	−0.011	0.070	0.148
Mothers’ SS → Fathers’ WtoFE → Fathers’ SWFaL	0.052	0.018	0.086	0.003**
Fathers’ SS → Mothers’ WtoFE → Mothers’ SWFaL	0.026	−0.005	0.057	0.106
Fathers’ SS → Fathers’ WtoFE → Mothers’ SWFaL	0.023	−0.002	0.049	0.077
Mothers’ SS → Mothers’ WtoFE → Mothers’ SWFaL	0.089	0.044	0.134	< 0.001***
Mothers’ SS → Fathers’ WtoFE → Mothers’ SWFaL	0.026	−0.002	0.053	0.065
Fathers’ SS → Mothers’ WtoFE → Adolescents’ SWFaL	0.009	−0.005	0.024	0.217
Fathers’ SS → Fathers’ WtoFE → Adolescents’ SWFaL	0.028	−0.001	0.056	0.059
Mothers’ SS → Mothers’ WtoFE → Adolescents’ SWFaL	0.032	−0.006	0.069	0.098
Mothers’ SS → Fathers’ WtoFE → Adolescents’ SWFaL	0.030	0.002	0.059	0.037*

SS, Perceived supervisor support; OJSS, Job satisfaction; SWFaL, Family satisfaction. *p < 0.05, **p < 0.01, ***p < 0.001.

The fathers’ WtoFE mediates the connection between their perceived supervisor support and family satisfaction. Additionally, the father’s WtoFE mediates the relationship between the mothers’ perceived supervisor support and the family satisfaction of both fathers and adolescents. In contrast, the mothers’ WtoFE only mediates the connection between their perceived supervisor support and family satisfaction. These results provide partial support for Hypothesis 8.

### 3.5 Testing differences between the within-domain and cross-domain paths

The relationship between WtoFE and job satisfaction did not show a significant difference when compared to the relationship between WtoFE and family satisfaction (Table 4) for both parents on an individual basis (*p* > 0.05). Similar results were found when comparing one parent’s WtoFE with the family and job satisfaction of the other parent, as well as in the relationship between each parent’s WtoFE and the adolescents’ family satisfaction. As a result, hypothesis 9 was not upheld.

**Table 4:** Difference between the within-domain and cross-domain perspectives paths.

Effects differences	*p*-value
(Mothers’ WtoFE → Mother’s OJSS) – (Mothers’ WtoFE→ Mothers’ SWFaL)	0.770
(Fathers’ WtoFE → Father’s OJSS) – (Fathers’ WtoFE→ Fathers’ SWFaL)	0.423
(Mothers’ WtoFE → Father’s OJSS) – (Mothers’ WtoFE→ Fathers’ SWFaL)	0.988
(Fathers’ WtoFE → Mother’s OJSS) – (Fathers’ WtoFE→ Mothers’ SWFaL)	0.128
(Fathers’ WtoFE → Adolescents’ SWFaL) – (Mothers’ WtoFE→ Adolescents’ SWFaL)	0.489

*WtoFE, Work-to-family enrichment; OJSS, Job satisfaction; SWFaL, Family satisfaction.

## 4 Discussion

This study evaluated the individual and interindividual relationships among perceived supervisor support, work-to-family enrichment (WtoFE), and satisfaction within Chilean dual-income families. By employing a mediation APIM, we identified how resources flow from the workplace to the family ecosystem, reinforcing Conservation of Resources (COR) theory. Our findings demonstrate that supervisor support initiates a “resource investment chain” that transcends individual boundaries, impacting both the employee and their family members. This research examines the dynamic between within-domain effects, where WtoFE bolsters satisfaction in the source domain (job satisfaction), and cross-domain effects, where these resources ripple into the family domain to enhance family satisfaction. By including adolescents in the analysis, we show that this “resource caravan” (Hobfoll, 2011) does not stop at the couple but extends to children, providing a systemic view of how workplace support functions as a primary engine for well-being across the work and family spheres.

### 4.1 Perceived supervisor support, job and family satisfaction, and work-to-family enrichment

According to COR theory, supervisor support acts as a critical contextual resource that minimizes the drain of personal energy, allowing employees to maintain a positive reservoir of resources (Hobfoll, 1989). Our findings confirm this logic at the individual level, as perceived support was directly associated with increased job satisfaction for both parents (H1a supported). This reinforces the role of the supervisor as a “resource gatekeeper” and is consistent with prior research demonstrating that supportive leadership is a robust predictor of workplace well-being (Burhanudin et al., 2020; Kalliath et al., 2020; Le et al., 2025; Öksüz et al., 2023; Otu et al., 2021).

However, this supportive resource did not directly translate into family satisfaction for either parent (H1b not supported), contrasting with some previous findings (Drummond et al., 2017). When considering the results for H1a and H1b together, our findings align with the affective path suggested by Greenhaus and Powell (2006). This path occurs when a resource generated in Role A promotes positive affect within that same role (Zhang et al., 2018). In this study, the resources generated by perceived supervisor support promoted positive affect specifically within the work domain—thereby enhancing job satisfaction—but did not directly spill over to family satisfaction. This indicates that these gains are primarily context-specific and remain within the work domain, requiring a transformative process—such as enrichment—to impact the family sphere.

Regarding crossover effects, fathers’ perceived supervisor support directly increased mothers’ job satisfaction (H2a partially supported). Theoretically, this relationship emerges through a crossover process where the resource gain of one individual increases their emotional availability, thereby enriching the partner’s resource pool (Bakker and Demerouti, 2013). As suggested by Booth-LeDoux et al. (2020), when fathers feel supported, their reduced stress allows partners to dedicate more of their own resources to their professional role, ultimately enhancing their job satisfaction (Soomro et al., 2018).

However, one parent’s supervisor support did not translate into increased family satisfaction for the partner (H2b not supported), challenging the idea that workplace support alone is sufficient to strengthen overall family functioning (Brady et al., 2021; Steiner and Krings, 2016). In contrast, fathers’ workplace resources did benefit the adolescents’ family satisfaction (H2c partially supported). This reinforces the “resource caravan” concept (Hobfoll, 2011), demonstrating that the positive environment initiated by a supervisor’s support can transcend the individual to benefit the family unit, particularly the well-being of adolescent children (Schnettler et al., 2022b).

Consistent with COR theory, individuals are motivated to acquire and protect resources to meet life’s demands (Hobfoll, 2001). Our findings support this “resource investment” logic, as supervisor support was positively associated with WtoFE for both parents (H3 supported). This confirms that a supportive supervisor provides a contextual resource that employees “re-invest” into their family role, acting as a catalyst for the enrichment process. This result aligns with meta-analytic evidence (Lapierre et al., 2018) and previous studies (Burhanudin et al., 2020; Carlson et al., 2019; Kalliath et al., 2020), reinforcing that workplace support is a universal and gender-neutral antecedent of enrichment.

Regarding crossover effects, our findings revealed that mothers’ perceived supervisor support was positively associated with fathers’ WtoFE (H4 partially supported). This aligns with the “resource caravan” logic (Hobfoll, 2011), suggesting that the support one partner receives creates a surplus of psychological capital that enhances the other partner’s ability to navigate their own work-family enrichment. While these results are consistent with the general crossover patterns identified by Brady et al. (2021) and Ho et al. (2013), the direction of the effect in our study is noteworthy. While Ho et al. (2013) found a crossover from husbands to wives, our data showed the inverse. A possible explanation for this difference is that resources available for transfer within the family may be limited or “specialized.” Since fathers’ support already crossed over to mothers’ job satisfaction and adolescents’ family satisfaction, this unique path from mothers to fathers suggests a gendered distribution of resources. It appears that the “resource caravan” initiated by the mother’s supervisor is specifically utilized to bolster the father’s enrichment process, highlighting a distinct inter-individual resource investment.

### 4.2 Work-to-family enrichment, along with satisfaction in both family and job roles

Our results confirm that WtoFE is a powerful driver of well-being, showing positive associations with job satisfaction (H5a supported) and family satisfaction (H5b supported) for both parents. This aligns with the “resource caravan” concept (Hobfoll, 2011), where resources gained at work initiate a positive cycle of gains across life domains. Specifically, the relationship with job satisfaction illustrates a within-domain effect, in which individuals feel more satisfied with their job because it is perceived as the source of family benefits (Amstad et al., 2011). In parallel, the link to family satisfaction reflects a cross-domain effect, in which the “psychological capital” acquired at work provides the necessary energy to enhance family interactions (Carlson et al., 2019). These findings are consistent with extensive meta-analytic and empirical evidence (Burhanudin et al., 2020; Landolfi et al., 2020; Lo Presti et al., 2020; Orellana et al., 2022; Xu et al., 2018; Zhang et al., 2018).

We anticipated that the resources accumulated through WtoFE would transfer between partners and to their adolescent children, creating a “resource reservoir” (Greenhaus and Powell, 2006). However, the expected interindividual within-domain effects were not found, as neither parent’s WtoFE influenced the other’s job satisfaction (H6a not supported). This differs from Liu and Cheung (2015), suggesting that when enrichment is derived specifically from supervisor support—rather than from general workplace resources—its impact may be too domain-specific to bolster the partner’s professional satisfaction.

Regarding the family domain, our results revealed a unidirectional crossover effect influenced by gender. Specifically, fathers’ WtoFE positively influenced the family satisfaction of both mothers (H6b partially supported) and adolescents (H6c partially supported), while no such transfer was observed from mothers. This aligns with the “resource caravan” logic (Hobfoll, 2011), where fathers’ workplace gains enhance the family’s collective energy. The lack of a reciprocal effect from mothers suggests that WtoFE may foster different personal resources for each parent (ten Brummelhuis and Bakker, 2012). For instance, enrichment might enhance positive mood in fathers, benefiting the family’s overall atmosphere, whereas in mothers, it may manifest as improved time management, primarily affecting their individual family experience (Schnettler et al., 2022a). These findings demonstrate that, while mothers’ enrichment remains personal, fathers’ WtoFE functions as a direct engine for the well-being of the broader family ecosystem. Notably, our study establishes a direct link between fathers’ WtoFE and the satisfaction of other family members, contrasting with previous research by Lo Presti et al. (2020), Matias and Recharte (2021), and Schnettler et al. (2022a), which suggested these relationships are primarily indirect and function through mediators such as work-family balance or the family atmosphere.

### 4.3 The role of work-to-family enrichment as a mediator

Our findings confirm that WtoFE acts as a bridge between workplace support and satisfaction, representing a “resource investment” chain in which initial contextual support provides the conditions for developing personal resources that are then reinvested in both life domains (Hobfoll, 2001). Regarding H7, this hypothesis was partially supported through three significant indirect pathways. At the individual level, perceived supervisor support from both parents positively influenced their own job satisfaction by enhancing their WtoFE, consistent with prior research (Burhanudin et al., 2020; Lu and Chang, 2014; Ollier-Malaterre et al., 2020). At the interindividual level, a mother’s positive perception of her supervisor was found to enhance the father’s job satisfaction through his WtoFE. This finding aligns with Carlson et al. (2019), who identified that the support one partner receives can affect the other’s professional commitment through the mediating role of enrichment.

Similarly, H8 was partially supported through four significant indirect pathways. At the individual level, WtoFE reinforced its role as a primary vehicle for transferring workplace support into personal family satisfaction, consistent with Carlson et al. (2019) regarding the mediation between supervisor support and marital satisfaction. Most notably, mothers’ perceived support initiated interindividual mediating roles that enhanced both fathers’ and adolescents’ family satisfaction via the fathers’ WtoFE. This underscores the “resource caravan” (Hobfoll, 2011), in which mothers’ workplace resources are “transferred” to bolster the father’s enrichment, which in turn benefits the entire family unit (Schnettler et al., 2022b). These complex indirect paths may explain why fathers and adolescents reported higher average family satisfaction than mothers, as they benefit from multiple resource streams within the family ecosystem.

In conclusion, our findings reveal a clear gender asymmetry in the accumulation of resources. Fathers received two positive indirect effects on both their job and family satisfaction—linked to both their own and their partners’ perceived supervisor support. In contrast, mothers only benefited from indirect effects stemming from their own perceptions. Although adolescents also received one indirect benefit, the overall pattern for parents shows a similar number of within-domain and cross-domain influences.

However, the comparison between the strength of these effects revealed no significant differences (H9 not supported). This contradicts previous studies and meta-analyses that favor the within-domain perspective (Lu and Chang, 2014; Xu et al., 2018; Zhang et al., 2018), which suggest that enrichment should impact the source domain (work) more robustly than the receiving domain (family). This lack of statistical difference may be due to the relatively weak strength of all significant pathways or the complexity of examining both approaches simultaneously across three family members. Similar results were reported by Schnettler et al. (2025) in a triadic study, suggesting that when the entire family ecosystem is considered, the traditional “resource proximity” logic may be balanced out by the multiple crossover and indirect paths that distribute resources across both domains.

### 4.4 Gendered dynamics in resource flow and crossover

The asymmetries observed in our APIM model suggest that the flow of resources within the family is deeply anchored in Gender Role Theory (Eagly and Wood, 2012). Our findings reveal that workplace resources are invested and received differently depending on the parent’s gender, reflecting traditional societal expectations. For instance, the fact that fathers’ perceived supervisor support directly enhances mothers’ job satisfaction and adolescents’ family satisfaction (H2a and H2c) suggests that support for the father—traditionally the primary provider—acts as a “systemic release.” By reducing the father’s professional stress, he becomes more emotionally available, which improves the family atmosphere and allows the mother to focus more effectively on her own career.

In contrast, the resources acquired by mothers appear to follow a “facilitator” pattern. Mothers’ supervisor support did not impact the partner’s satisfaction directly but instead fueled the father’s enrichment process (H4 and H7). This indicates that, in this context, mothers may reinvest their workplace gains into supporting the father’s ability to balance his roles. Finally, the finding that only fathers’ WtoFE directly boosts the family satisfaction of others (H6) underscores an asymmetric valuation of enrichment; while a father’s success in balancing work and family is perceived as a collective gain for the ecosystem, a mother’s enrichment remains a more individualized resource. Thus, the “resource caravan” in Chilean dual-income families is not gender-neutral but is filtered through roles in which the father’s well-being serves as the primary engine of the family’s satisfaction, while the mother’s support acts as a crucial yet indirect catalyst for the system.

### 4.5 Theoretical and practical implications

#### 4.5.1 Theoretical implications

Our research enhances the “resource caravan” framework (Hobfoll, 2011) by positioning WtoFE as a critical interindividual bridge. We demonstrate that enrichment is the essential mechanism through which supervisor support transcends the individual employee to benefit the entire family ecosystem. By showing that these indirect effects enhance job satisfaction for the partner and family satisfaction for both the partner and adolescent children, we redefine WtoFE as a collective process rather than a personal one. This highlights its role as a systemic connector that sustains well-being across both work and family domains.

Second, regarding the relative impact of enrichment on satisfaction, we found no significant difference between the strength of within-domain effects (WtoFE → job satisfaction) and cross-domain effects (WtoFE → family satisfaction), leading to the rejection of H9. This result challenges the traditional “resource proximity” logic, which posits that the affective reaction toward the domain of origin (work) should result in a stronger within-domain association. Our findings suggest that in complex triadic systems, the benefits of enrichment are distributed more evenly across life domains than previously theorized, possibly because the systemic value of resources at home balances the individual affective attribution toward the workplace.

Finally, we contribute to Crossover Theory by identifying gender-based asymmetries in resource flow. Our findings suggest that mothers and fathers occupy different roles in the resource investment chain: mothers often act as “resource facilitators” (initiating the crossover), while fathers’ enrichment functions as the “systemic engine” that directly impacts the well-being of all family members.

#### 4.5.1 Practical implications

From a management perspective, the pervasive impact of parents’ perceived supervisor support on the entire family unit emphasizes that organizational support is a public health asset. Organizations should move beyond general family-friendly policies toward specific supervisor training programs. These programs should focus on “supportive leadership” behaviors—such as active listening, recognition of achievements, and fostering emotional well-being—as these are the primary catalysts for the enrichment process in both parents.

Specifically, companies must recognize that supporting fathers directly enhances the well-being of the broader family ecosystem, including adolescents. Simultaneously, supporting mothers has a crucial indirect ripple effect, stabilizing dual-income households by bolstering the partner’s resources. By fostering a supportive work environment for both genders, organizations not only improve individual job satisfaction but also contribute to a positive resource caravan that reduces turnover and increases overall systemic performance. Investing in supervisor support is, therefore, a strategic investment in the stability and quality of life of the contemporary family.

### 4.6 Limitations and future research

Despite its contributions, this study has several limitations that open new avenues for research. The effects identified through APIM and SEM should not be interpreted as causal relationships, as the research’s cross-sectional design precludes causal verification. Therefore, longitudinal, experimental, or quasi-experimental studies are necessary to establish causality. It is also important to note that testing a mediation framework with single-timepoint data can be sensitive to (CMB). Although statistical checks performed on our data indicate that CMB does not significantly threaten the validity of the results, the lack of temporal separation between variables remains a limitation. Future research should employ longitudinal designs to more accurately capture the dynamic and sequential process of work-to-family enrichment.

Second, the use of a non-probabilistic sampling method restricts the capacity to extrapolate the findings to the broader population of dual-income parents with adolescents in Chile. This approach introduces potential selection biases; for instance, self-selection bias may arise, as families who voluntarily agreed to participate might have a greater interest in the study’s topics than those who declined. Furthermore, since data collection relied on online questionnaires, the sample may be biased toward families with higher digital literacy and consistent internet access. Additionally, the sample focused on families with adolescents between the ages of 10 and 15. Future research should employ a probabilistic sampling approach to mitigate these biases, enhance the applicability of the findings to a wider demographic, and investigate how perceived supervisor support affects the well-being of older adolescents. Because family socioeconomic status and the ages of family members played a role in influencing the results as control measures, future research should investigate these elements as moderators. This would allow for a better understanding of how socioeconomic conditions or different adolescent developmental stages influence the strength of the observed crossovers.

Third, the absence of explicit attention checks or formal detection techniques for responses indicating insufficient effort is another limitation. Although procedural measures—such as monetary compensation and detailed instructions—were implemented to promote engagement, the risk of inattentive responding cannot be fully excluded. This should be considered when interpreting the results, as low-effort responses may affect data precision. Future research should incorporate clear attention checks and statistical measures to identify insufficient effort in responses, thereby enhancing data quality.

Fourth, because this study was conducted in a single city of a developing Latin American country with conventional family structures, there is a need for cross-cultural research that includes nations or societies with different degrees of gender equality. Future studies should also explore the absence of statistical differences between within-domain and cross-domain perspectives (H9) found here, as this contradicts established meta-analytic trends.

Fifth, our data did not address the parents’ specific working milieu or other workplace resources beyond supervisor support. Future research should examine whether coworker support or organizational culture produces similar triadic effects. Finally, qualitative research is needed to explore the specific personal resources developed by parents and to understand why the resource caravan in this context appears to be initiated primarily by mothers’ support and mediated through fathers’ WtoFE. Understanding these underlying mechanisms will provide a deeper, more nuanced view of the gendered asymmetries identified in this study.

## 5 Conclusion

This study concludes that supervisor support is a fundamental driver of well-being that transcends the professional sphere to nurture the family ecosystem. By applying an APIM framework to Chilean dual-income families, we demonstrated that work-to-family enrichment is the essential link through which workplace resources are transformed into job and family satisfaction for parents and adolescents alike. Our findings highlight a significant gender asymmetry: while both parents benefit from support, the pathways through which these resources flow are specialized, with fathers’ enrichment acting as a direct catalyst for family satisfaction and mothers’ support serving as an interindividual facilitator.

Ultimately, this research underscores the need for labor and family policies that recognize the interdependence of work and home life. Policymakers and organizations must collaborate to foster supportive work environments, not merely as a benefit for the individual employee, but as a strategic investment in the stability and quality of life of the next generation.

## Data Availability

The original contributions presented in the study are included in the article/Supplementary material, further inquiries can be directed to the corresponding author.
