# Supplementary material for: Parents’ perceived supervisor support and work-to-family enrichment affect job and family satisfaction in dual-income parents and their adolescents
Source: Front Psychol. 2026 May 19;17:1694200. doi: 10.3389/fpsyg.2026.1694200 (PMC13226111; doi:10.3389/fpsyg.2026.1694200)
Supplement: Supplementary file 2 [file Table_1.DOCX]

**Supplementary material**

**Items of the Perceived Supervisor Support Scale**

1. My supervisor is concerned about the welfare of those under him or her.

2. My supervisor pays attention to what I’m saying.

3. My supervisor exposes me to hostility and conflict (reversal).

4. My supervisor is helpful in getting the job done.

5. My supervisor is successful in getting people to work together.

6. My supervisor gives me credit for things I do well.

7. My supervisor criticizes me for small things (reversal).

8. My supervisor backs me up if there is a problem.

9. My supervisor cares about me.

10. My supervisor appreciates me
